# Correction to: *BMC Public Health 300 & 712*

**DOI:** 10.1186/s12889-024-19026-3

**Published:** 2024-06-07

**Authors:** Sian Reece

**Affiliations:** Hull York Medical School, York, North Yorkshire UK

**Correction to:** ***BMC Public Health*** **24, 300 (2024).** 10.1186/s12889-024-17773-x **&** ***BMC Public Health*** **24, 712 (2024).** 10.1186/s12889-024-18226-1

The original publications of the below articles both contained an erroneous funding section. The incorrect and correct information is listed below.

**Incorrect**

- 10.1186/s12889-024-18226-1
  - SR is in receipt of a National Institute for Health and Care Research (NIHR) Doctoral Research Fellowship (grant number NIHR300677). The views expressed in this publication are those of the authors and not necessarily those of the NHS, the NIHR or the Department of Health and Social Care.
- 10.1186/s12889-024-17773-x
  - SR is in receipt of a National Institute for Health and Care Research (NIHR) Doctoral Research Fellowship (grant number NIHR300677). The views expressed in this publication are those of the authors and not necessarily those of the NHS, the NIHR or the Department of Health and Social Care.

**Correct**

- 10.1186/s12889-024-18226-1
  - SR is in receipt of a National Institute for Health and Care Research (NIHR) Doctoral Research Fellowship (grant number NIHR300677). The views expressed in this publication are those of the authors and not necessarily those of the NHS, the NIHR or the Department of Health and Social Care. The Born in Bradford’s Better Start (BiBBS) cohort has received funding from the Big Lottery Fund as part of the A Better Start programme. The Big Lottery Fund have not had any involvement in the design or writing of the paper. This research is part of the Healthy Children Healthy Families Theme of the National Institute for Health Research (NIHR) Collaboration for Leadership in Applied Health Research and Care (CLAHRC) Yorkshire and Humber. The views expressed in this publication are those of the authors and not necessarily those of the NHS, the NIHR or the Department of Health. This research also forms part of the ActEarly UK Prevention Research Partnership Consortium (https://ukprp.org/what-we-fund/actearly/) (MR/S037527/1). The views expressed in this publication are those of the authors and not necessarily those of the NHS, the NIHR, the Department of Health or the funders.
- 10.1186/s12889-024-17773-x
  - SR is in receipt of a National Institute for Health and Care Research (NIHR) Doctoral Research Fellowship (grant number NIHR300677). The views expressed in this publication are those of the authors and not necessarily those of the NHS, the NIHR or the Department of Health and Social Care. This research is part of the Braford Inequalities Research Unit, funded through the Reducing Inequalities in Communities programme commissioned by Bradford City Clinical Commissioning Group (now Bradford and District and Craven Health and Care Partnership). This research also forms part of the ActEarly UK Prevention Research Partnership Consortium (https://ukprp.org/what-we-fund/actearly/) (MR/S037527/1). The views expressed in this publication are those of the authors and not necessarily those of the NHS, the NIHR, the Department of Health or the funders.

